# Silver Decorated βTCP-Poly(3hydroxybutyrate) Scaffolds for Bone Tissue Engineering

**DOI:** 10.3390/ma14154227

**Published:** 2021-07-28

**Authors:** Joanna Czechowska, Szymon Skibiński, Maciej Guzik, Aneta Zima

**Affiliations:** 1 Department of Ceramics and Refractories, Faculty of Materials Science and Ceramics, AGH University of Science and Technology, Mickiewicza Av. 30, 30-059 Krakow, Poland; azima@agh.edu.pl; 2 Jerzy Haber Institute of Catalysis and Surface Chemistry, Polish Academy of Sciences, Niezapominajek 8, 30-239 Krakow, Poland; maciej.guzik@ikifp.edu.pl

**Keywords:** β tricalcium phosphate, silver-decorated scaffolds, P(3HB) coating, P(3HB) degradation, bacterial polymer

## Abstract

Implantations in orthopedics are associated with a high risk of bacterial infections in the surgery area. Therefore, biomaterials containing antibacterial agents, such as antibiotics, bactericidal ions or nanoparticles have been intensively investigated. In this work, silver decorated β tricalcium phosphate (βTCP)-based porous scaffolds were obtained and coated with a biopolymer—poly(3-hydroxybutyrate)-P(3HB). To the best of our knowledge, studies using silver-doped βTCP and P(3HB), as a component in ceramic-polymer scaffolds for bone tissue regeneration, have not yet been reported. Obtained materials were investigated by high-temperature X-ray diffraction, X-ray fluorescence, scanning electron microscopy with energy dispersive spectroscopy, hydrostatic weighing, compression tests and ultrahigh-pressure liquid chromatography with mass spectrometry (UHPLC-MS) measurements. The influence of sintering temperature (1150, 1200 °C) on the scaffolds’ physicochemical properties (phase and chemical composition, microstructure, porosity, compressive strength) was evaluated. Materials covered with P(3HB) possessed higher compressive strength (3.8 ± 0.6 MPa) and surgical maneuverability, sufficient to withstand the implantation procedures. Furthermore, during the hydrolytic degradation of the composite material not only pure (R)-3-hydroxybutyric acid but also its oligomers were released which may nourish surrounding tissues. Thus, obtained scaffolds were found to be promising bone substitutes for use in non-load bearing applications

## 1. Introduction

Tissue engineering is a technique that involves the in vitro seeding and attachment of cells onto a three-dimensional scaffold. In the case of bone tissue engineering, investigations have been focused mostly on synthetic bioceramic scaffolds based on calcium phosphates, such as hydroxyapatite or tricalcium phosphate. Due to their chemical similarity to an inorganic component of bone, hydroxyapatite, as well as βTCP- and αTCP-based materials, show excellent biocompatibility and osteoconductivity. To further enhance their physicochemical and biological characteristics modifications with monovalent (Ag^+^, Na^+^), divalent (Mg^2+^, Zn^2+^) and trivalent (Fe^3+^) metal ions were done [1,2,3]. Due to the high risk of bacterial infections during implantation procedures, biomaterials containing antibacterial agents, such as antibiotics, bactericidal ions (e.g., Ag^+^, Au^+^, Cu^2+^) and nanoparticles have been intensively investigated. Recently, silver-modified biomaterials have been prepared by various methods that, *inter alia*, involve the incorporation of silver ions into their structure or attachment of silver nanoparticles on their surface. Silver-containing tricalcium phosphate microspheres composed of α/βTCP phases were synthesized using an ultrasonic spray-pyrolysis technique [4]. Su et al. [5] studied silver containing calcium phosphate coatings on pure iron foam obtained via co-deposition and post-treatment method. Siek et al. [6] used wet chemical method to obtain bactericidal αTCP-based bone cements with silver-modified hydroxyapatite (Ag-HA) and CaCO_3_. It has been found that bone cement matrix did not impede the Ag^+^ ions release from the Ag-HA agglomerates and antibacterial activity depended on the kind of bacterial strain. Hoover et al. [7] obtained silver-modified porous β-tricalcium phosphate (βTCP) scaffolds using liquid porogen based method. In their work silver added in the amount between 0.5 and 2 wt.% Ag_2_O could be released over a long period of time without compromising the biocompatibility of the scaffolds. The bactericidal activity was achieved due to sustained release of Ag^+^ ions through the continuous dissolution of Ag-modified βTCP. Materials for various biomedical applications, including chitosan [8], cellulose and its derivatives [9,10] as well as mesoporous carbons [11] decorated with silver nanoparticles were also examined. However, silver decorated calcium phosphate-based scaffolds seem to be an interesting and not yet fully explored area.

Calcium phosphate (CaP)-based bioceramic scaffolds are inherently brittle and often cannot match the mechanical properties of the bone. Thus, composites made of CaPs and bioresorbable polymers have also been investigated [12,13]. Recently, many researchers have been working on the fabrication of polymer-coated bone scaffolds including ceramic-biopolymer hybrid systems [14,15]. A new trend in the development of biomedical composites is a usage of polyhydroxyalkanoates (PHAs)—the bacterial derived polymers [16,17]. Under normal growth conditions, most bacteria produce only a small amount of PHA (1–15%). When special growth conditions and fermentation strategies are applied, the synthesized PHA can reach almost 90% [18]. Medical applications require a constant and reproducible quality of PHAs, which can be achieved via bacterial production in a rigorous culture [19]. Polyhydroxyalkanoates are a diverse group of materials with different applications and properties. It is known that more than 150 types of PHAs can be synthesized by microorganisms [20]. The material characteristics associated with PHAs are affected by many parameters including their chemical structure and type of monomer units with molecular mass. PHAs can be classified into short chain length (scl) PHAs with 4–5 carbons in pending monomers backbone or medium chain length (mcl) PHAs with (6–14 carbon atoms), when aliphatic monomers are present, and may further be grouped a homo-polymers (either scl-PHAs or mcl-PHAs) or copolymers (a mixture of different monomers of scl-PHA and/or mcl-PHA monomers). There were many attempts to use PHAs in medical applications due to their excellent biocompatibility and biodegradability [21,22,23,24]. PHAs have an advantage over other bioplastics such a poly(lactic acid) (PLA) or poly(D,L-lactide-co-glycolide) (PLGA) since their monomers (3-hydroxylated acids) are quickly metabolized within the human body. Furthermore, they can be naturally detected in almost all parts of the body as a degradation product and were found not to cause carcinogenesis during long-term implantation [25,26]. Among the PHA family, the P(3HB) is the most common and well-characterized scl-PHA. The possibility of using P(3HB) as coating materials for composite type inorganic-organic scaffolds was demonstrated among others by Montazeri et al. [27]. It has been found that scaffold made of bioglass covered with polyhydroxybutyrate (PHB) possessed higher mechanical strength and bioactivity than nano-bioglass strut alone. In this study, to improve the mechanical properties and surgical maneuverability of brittle silver decorated βTCP scaffolds, they were covered with bioresorbable P(3HB) polymer. These kinds of highly-porous materials may be potentially applied for filling small bone defects in non-load or low-load bearing places. In the future the P(3HB) coating may serve as a drug delivery vehicle. Development of novel methodologies to fabricate bioceramic-PHAs composites may open new horizons for their applications in medicine. Therefore, silver decorated calcium phosphates, covered by a PHA layer seem to be interesting materials that have not yet been explored. This proof-of-concept study delivers some insights into the synthesis of such materials and their characterization, setting a benchmark for further developments of material for regenerative medicine based on biopolymers and bioceramics.

## 2. Materials and Methods

### 2.1. Tricalcium Phosphate (TCP) Powder Preparation

The initial βTCP and Ag-βTCP powders were synthesized via modified wet precipitation method. Briefly, Ca(OH)_2_ (POCH, Gliwice, Poland) and H_3_PO_4_ (POCH, Gliwice, Poland) were applied as substrates (Ca/P = 1.5). Silver nitrate (AgNO_3;_ Chempur, Piekary Śląskie, Poland) was used as the source of silver. The amount of silver was equal 1.0 wt.%. The synthesis was done in a room temperature and the pH of the reaction mixture was maintained between 5 and 7. Afterwards, the precipitate was left to mature (48 h), centrifuged (Multifuge 3L, Thermo Heraeus Kendro, Osterode, Germany) and dried. The produced powder was ground in a ball mill (PM100, Retsch, Haan, Germany) and calcined for 2 h at 900 °C (Nabertherm L32/14, Nabertherm GmbH, Lilienthal, Germany). Finally, powders were grounded in an attritor mill (designed at AGH-UST, Krakow, Poland) and sieved (<63 µm). The specific surface area of the initial βTCP and Ag-βTCP powders was determined by the Brunauer−Emmett−Teller (BET) method using nitrogen (ASAP 2000, Micrometric Instruments Inc., Norcross, GA, USA) and was equal 6.32 ± 0.01 and 7.85 ± 0.03 m^2^/g, respectively.

### 2.2. Preparation of the Bioceramic Scaffolds

Scaffolds were prepared via the polyurethane sponge replica technique according to the previously described procedure [14]. Briefly, cubic-shape polyurethane templates (Bulpren S 28089, 10 mm edge length, Recticel Flexible Foams, Wetteren, Belgium) were immersed into the ceramic slurry to get a complete impregnation and slightly squeezed to remove the slurry excess. The slurry composed of Ag-βTCP ceramic powder, distilled water, methylcellulose (Fluka, Buchs, Switzerland) and Dispex^®^ AA4040 as a dispersant agent (BASF, Arnhem, Netherlands). After impregnation, all specimens were dried, and heat treated at 1150 or 1200 °C.

### 2.3. P(3HB) Synthesis and Purification

Polyhydroxybutyrate (P(3HB)) was produced by bacterial fermentation from glycerol (Orlen Południe S.A., Trzebinia, Poland) in the presence of NaCl (Chempur, Piekary Śląskie, Poland) at 45 °C using bacterial strain *Zobellella denitrificans* (Wilhelms-Universität Münster, Germany) as described in [28]. The biomass after fermentation was lyophilized and then extracted with chloroform (Chempur, Piekary Śląskie, Poland). The resulting solution was passed through activated charcoal (Merck, Warsaw, Poland) and 0.2 µm polytetrafluoroethylene (PTFE) filter (Avantor, Gdańsk, Poland). Next, the P(3HB) solution was concentrated on a rotatory evaporator (Heidolph Hei-VAP Industrial B, Heidolph Instruments GmbH & Co. KG, Schwabach, Germany), precipitated in an ice-cold methanol solution (Chempur, Piekary Śląskie, Poland) and dried in an oven (Binder FED400, Binder GmbH, Tuttlingen, Germany). The cleaned-up polymer was reconstituted in chloroform (5% *w*/*v*) for further experiments.

### 2.4. Coating of the Bioceramic Scaffolds with P(3HB)

**Figure 1:**
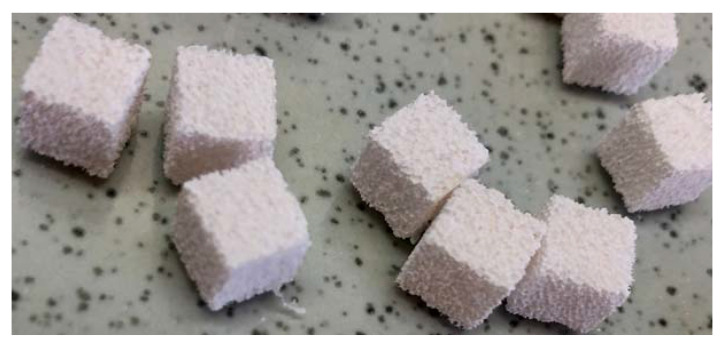
Bioceramic scaffolds coated with poly(3-hydroxybutyrate) (P(3HB)).

To cover the bioceramic scaffold with polymer, ceramic specimens were infiltrated with P(3HB) chloroform solution. After that, all samples were dried at room temperature for 7 days and subjected to further studies (Figure 1). The composition of materials is presented in Table 1.

### 2.5. Chemical and Phase Composition 

To determine the chemical composition of materials, the X-ray fluorescence method (XRF) was applied (WDXRF Axios Max, PANalytical, Malvern, UK). The phase composition of the initial powder was analyzed via High-Temperature X-Ray Diffraction (HT-XRD) (X’Pert Pro, Malvern Panalytical, Malvern, UK) and the phase composition of scaffolds was analyzed via powder X-ray diffraction method (D2 Phaser diffractometer, Bruker, Billerica, MA, USA). The intensity was recorded within the 2θ range from 10° to 40°, at 0.02° intervals and a scanning speed of 2.5°·min^−1^. Crystalline phases were identified by comparing the experimentally obtained diffractograms to the Joint Committee on Powder Diffraction Standard: βTCP (JCPDS 00-055-0898) and calcium deficient hydroxyapatite—CDHA (JCPDS 09-043). Rietveld refinement of the XRD patterns was performed with DIFFRAC SUITE TOPAS software (Version 4.2.0.1, 2011, Bruker, Billerica, MA, USA).

### 2.6. Microstructure

The microstructure of obtained scaffolds was assessed using a scanning electron microscope (Nova NanoSem 200, FEI Company, Hillsboro, OR, USA). Chemical composition in microareas was analyzed via an energy-dispersive X-ray spectroscopy microanalyzer (EDX, Oxford Instruments, Oxford, UK). Before SEM observations all samples were coated (Agar Sputter Coater 108, Stansted, Essex, UK) with a thin layer of gold (Mennica-Metale, Radzymin, Poland).

### 2.7. Porosity Studies

The total, open and closed porosities of the scaffolds were determined by hydrostatic weighing based on the Archimedes’ principle. All measurements were made in sextuple.

### 2.8. Compressive Strength Measurements

The scaffolds were subjected to compression tests using the universal testing machine (Instron 3345, Norwood, MA, USA) with a crosshead displacement speed of 1.0 mm·min^−1^. The compression test was carried out on 15 cubic specimens for each material (with the edge length of about 8 mm).

### 2.9. Degradation Studies and UHPLC-MS Measurements

Composite materials were incubated in distilled water at 37 °C in polypropylene (PP) containers (Avantor, Gdańsk, Poland). Liquid samples (1 mL) of incubated specimens were filtered and analyzed on Agilent 1290 Infinity System (Agilent, Santa Clara, CA, USA) with automatic autosampler and MS Agilent 6460 Triple Quad Detector (Agilent, Santa Clara, CA, USA) equipped with Agilent Zorbax Eclipse Plus C18 column (2.1 mm × 50 mm, 1.8 µm) as described previously [13]. Briefly, samples were developed on the column at 30 °C at a flow rate of 0.5 mL·min^−1^ and with gradient elution of solvent A (0.1% *v*/*v* formic acid in water, Witko, Łódź, Poland) and solvent B (0.1% *v*/*v* formic acid in acetonitrile, Witko, Łódź, Poland) as follows: 0.00 min (50% A/50% B) to 1.90 min (10% A/90% B) to 1.91 min (50% A/50% B) to 2.60 min (50% A/50% B). The injection interval was 2.6 min. MS Agilent 6460 Triple Quad tandem mass spectrometer (Agilent, Santa Clara, CA, USA) with Agilent Jet Stream ESI interface was used in negative ion mode. Nitrogen was applied as the drying gas and for collision-activated dissociation (a flow rate: 10 L·min^−1^). Drying gas and sheath gas temperatures were set to 350 °C. Capillary voltage was equal to 3500 V, whereas the nozzle voltage was 500 V. The elution profiles were monitored in a scan range of 50–1000 *m*/*z* first to determine main peaks. Next, main degradation compounds were monitored in multiple reaction monitoring mode (MRM) with the transitions, polarity, fragmentor (F) and collision energies (CE) presented in Table S1 (Supplementary Material). Workstation MassHunter Data Acquisition 1.1 version (Agilent, Santa Clara, CA, USA) was used for ultrahigh-pressure liquid chromatography with mass spectrometry (UHPLC-MS) system control, data acquisition, and data processing.

### 2.10. Statistical Analysis

Statistical analysis was carried out applying one-way analysis of variance (ANOVA) and post hoc Tukey HSD multiple comparisons.

## 3. Results and Discussion

### 3.1. Chemical and Phase Composition

The XRF analysis demonstrated that the 1.097 wt.% of silver was introduced during the synthesis of the initial Ag-βTCP powder (Table 2). Furthermore, the X-ray fluorescence measurements confirmed the presence of silver in the scaffolds after sintering in both 1150 and 1200 °C. It has been shown that the procedure of scaffold preparation allowed to retain the desired amount of silver in the final material (1.227–1.259 wt.%).

**Figure 2:**
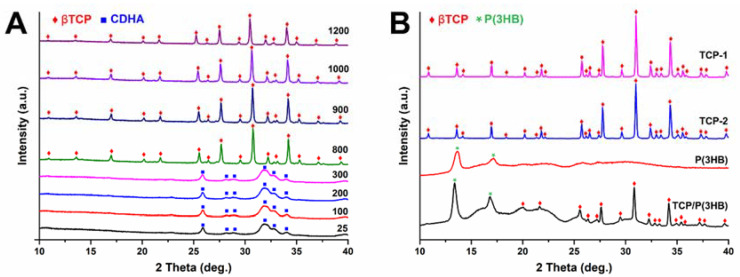
The High-Temperature X-ray Diffraction (HT-XRD) patterns of (**A**) Ag-βTCP powder measured in temperatures between 25 and 1200 °C and the XRD patterns of (**B**) TCP-1, TCP-2 scaffolds as well as polymer P(3HB) and coated composite TCP/P(3HB).

The XRD analyses of initial powders as well as sintered scaffolds were performed to determine possible changes in the phase composition induced by the sintering process (Figure 2).

HT-XRD analysis (Figure 2A) of the non-calcinated Ag-βTCP powder indicated that at the room temperature of 25 °C up to 300 °C powder consists of calcium-deficient hydroxyapatite (CDHA). These results are consistent with Ghosh et al. [29] who observed the existence of CDHA phase of the undoped βTCP powder prepared by coprecipitation method up to 600 °C. Ag-βTCP powder when calcined from 800 to 1200 °C transforms into crystalline βTCP.

An examination of X-ray diffractograms revealed that when sintered at 1150 and 1200 °C scaffolds consist of only one crystalline phase, i.e., βTCP (Figure 2B). According to Ryu et al. [30], pure βTCP ceramics sintered at 1200 °C gradually decompose to αTCP. Skibiński et al. [15] conducting research on bioceramics βTCP-based scaffolds also observed the partial phase transformation of βTCP to αTCP at 1200 °C. The results of our studies indicate that modification of β-tricalcium phosphate with silver led to stabilization of βTCP phase. This finding stayed in agreement with results obtained by Gokcekaya et al. [31,32] who stated that the addition of Ag in CaPs stabilized the βTCP phase after sintering. The XRD showed that the layer of P(3HB) is semicrystalline with clearly displayed peaks at 2θ = 13.63° and 17.19°. The diffractogram of the polymer-coated composite reveals the presence of reflexes from βTCP and P(3HB) as well as the amorphous halo originated from the polymer.

Furthermore, it has been shown that the lattice parameter c of the silver modified βTCP decreased from 37.3988 Å (βTCP) to 37.3952 Å (Ag-βTCP), while parameter a slightly increased from 10.4346 Å (βTCP) to 10.4360 Å (Ag-βTCP). Similar observations were done by Gokcekya et al. [31,33], who stated that the βTCP crystal structure consists of columns A and B, having different sites of Ca, P, and O atoms located along the c-axis. In the βTCP structure, Ca(4) sites, including vacancies, may be occupied by the silver ions. This may result in a decrease in the lattice parameter c and stabilization of the βTCP structure. The existence of silver in Ca(4) positions in Ag-doped βTCP was recently confirmed via the high-angle annular dark-field scanning transmission electron microcopy observations [34].

### 3.2. Microstructure

**Figure 3:**
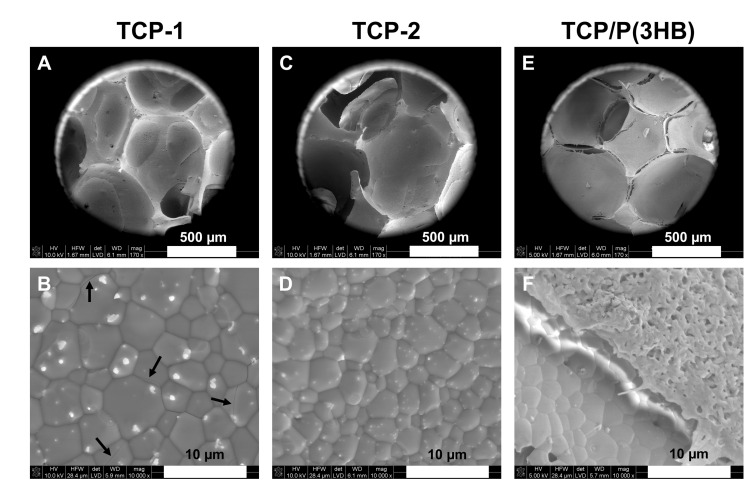
SEM microphotographs of scaffolds: TCP-1 (**A**,**B**), TCP-2 (**C**,**D**) and TCP/P(3HB) (**E**,**F**). Arrows indicate local intergranular cracks.

SEM observations of the obtained scaffolds demonstrated macroporous microstructure with pore sizes between 100 and 700 µm and a predominance of pores around 300–500 μm. The pore sizes from this range are recognized as appropriate for bone tissue ingrowth [35,36]. Furthermore, the well-densified pore walls were observed. The densification behavior of scaffolds can be considered as a two-stage phenomenon, characterized by (I) an early onset and a low densification rate and (II) a sharp increase in the densification rate at higher temperatures. In Figure 3 extensive grain growth is observed, especially in the samples sintered at 1200 °C. The uncontrolled increase in the size of βTCP grains can be unfavorable, as materials with grains larger than the critical size do microcrack. In the case of material TCP-1, exceeding a critical grain size (average grain size 5.3 ± 1.3 µm) resulted in regions with local intergranular cracks (Figure 3B). Therefore, scaffolds sintered at temperature 1150 °C (TCP-2 with an average grain size 3.2 ± 0.8 µm) were the material of choice for coating with P(3HB).

Furthermore, silver particles of sizes between 624 ± 139 nm and 199 ± 37 nm for TCP-1 and TCP-2, respectively, were observed on the surfaces of scaffolds after sintering (Figure 3). It is possible that during the wet chemical synthesis silver was not entirely introduced into the structure of βTCP. Simultaneously, during the synthesis, chemical reaction between Ag^+^ and OH^−^ ions occurred and silver oxide was produced, according to Equation (1). As a result of silver oxide formation, the color of the reaction mixture during the synthesis changed into brownish.

2Ag^+^ + 2OH^−^ → Ag_2_O_(s)_ + H_2_O (1)

The amount of silver oxide was too small to detect it with XRD measurements. The thermal treatment of precipitate caused further changes in the composition of scaffolds. Solid silver oxide decomposes at temperatures above 300 °C, yielding metallic silver and oxygen gas.

2Ag_2_O_(s)_ = 4Ag_(s)_ + O_2(g)_ (2)

**Figure 4:**
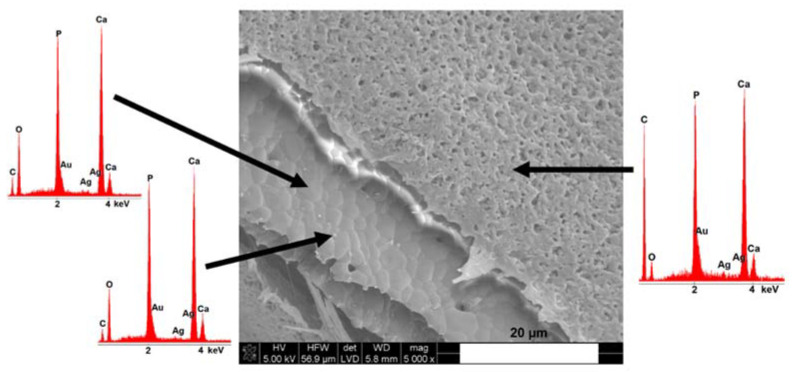
SEM microphotograph and energy-dispersive X-ray spectroscopy (EDS) analysis of the scaffold covered with poly(3hydroxybutyrate) (TCP/P(3HB)).

According to Waterhouse et al. [37], the transformation of silver (I, III) oxide AgO to Ag_2_O occurs with heating in the 100–200 °C region, while the complete thermal decomposition of the Ag_2_O to metallic silver and O_2_ occurs at 400 °C. The presence of silver particles on the surfaces of scaffolds was confirmed by SEM-EDX analysis (Figure 4). The formation of metallic particles instead of a film is usually observed if at the temperature of the process, the reactant is in the liquid phase (the melting point of silver is 961 °C) [38]. In our studies, the sintering temperatures exceeded the melting point of silver, however, they were below its boiling point (2162 °C). Thus, during the heat treatment of scaffolds silver was in the liquid state. The mechanism of silver nucleation was most probably through condensation of supersaturated vapor of the low-volatile product. The time taken to form the first nuclei on the surface of the reactant (so-called induction period) could have been shortened due to the presence of CaPs grains or impurities. Similar observations were done by Gokcekaya et al. [31], who stated the presence of silver particles on the surfaces of calcium phosphate-based materials after high-temperature thermal treatment. Since the growth of particles is kinetically controlled, the particle size increases with the time of heat treatment [39].

Figure 3E,F and Figure 4 show the SEM microphotographs of scaffolds coated with P(3HB). The P(3HB) coating was microporous and characterized by a surface with roughness in the submicrometer range. Moreover, the presence of the polymer layer on the TCP/P(3HB) surface (Figure 4) was confirmed by the appearance of carbon atoms via energy-dispersive X-ray spectroscopy (EDS) analysis. The similar, thin (10 ± 2 µm), porous poly(3-hydroxybutyrate) film was observed by Peschel et al. [40] as well as Wang et al. [41]. SEM observations revealed that the P(3HB) film presented a good adhesion to the surface of scaffold. However, the P(3HB) is not ductile and therefore probably its adhesion to TCP could be improved by producing coating with a higher degree of flexibility.

### 3.3. Porosity Studies

The open porosity of scaffolds, determined by Archimedes method, was 66.7 ± 2.7 vol%, 68.1 ± 4.6 vol% and 61.8 ± 3.0 vol% for TCP-1, TCP-2 and TCP/P(3HB) scaffolds, respectively (Table 3). There is a significant change in the closed porosity of the materials after infiltration with the polymer. The closed porosity of TCP/P(3HB) (10.1 ± 3.0 vol%) increased when compared to uncoated scaffolds. This phenomenon may relate to the clogging of smaller pores by P(3HB). Similar observations were done by Cichoń et al. [14] in the case of tricalcium phosphate/poly(3-hydroxyoctanoate) (TCP/PHO) scaffolds. There is a significant change in the total porosity of the materials after infiltration with the polymer. The closed porosity increased c.a. three times for material TCP/P(3HB) (10.1 ± 3.0 vol%) when compared to uncoated scaffolds. Nevertheless, scaffolds of similar porosity, as well as size and shape of pores, have been reported to be optimal for bone tissue engineering, so produced materials seem to be promising candidates for medical applications [42]. Highly porous tricalcium phosphate-based scaffolds, with interconnected, spherical pores may provide a biocompatible surface for adhesion and proliferation of cells and newly formed bone tissue ingrowth, as well as new blood vessels development.

### 3.4. Compressive Strength Measurements

**Figure 5:**
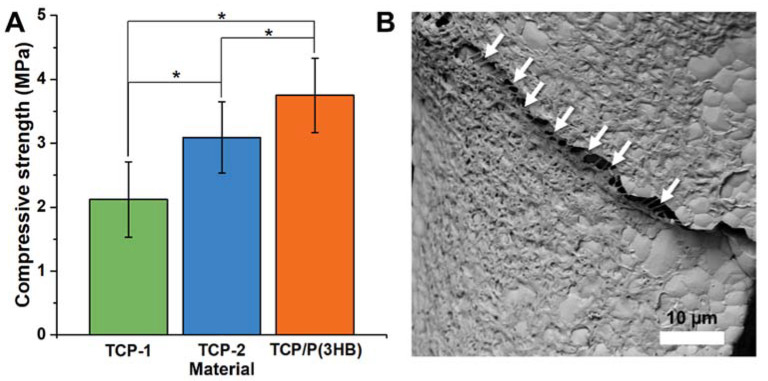
The compressive strength of the scaffolds (**A**) TCP-1, TCP-2, TCP/P(3HB) and (**B**) SEM microphotograph of TCP/P(3HB) after compressive test. Arrows indicate polymeric microfilaments connecting ceramics’ cracks. Statistically significant differences were indicated by * *p* ≤ 0.01.

In general, the compressive strength of a scaffold depends on its macroporosity, pore size distribution and geometry, as well as the intrinsic strength of the strut. The results of compressive tests showed that Ag-βTCP scaffolds sintered with 1200 °C (TCP-1; 2.1 ± 0.6 MPa) possessed lower compressive strength than those sintered in 1150 °C (TCP-2; 3.1 ± 0.6 MPa) (Figure 5). All observed differences were statistically significant (*p* < 0.01). The open and close porosity of both materials have comparable differences in mechanical strength which are most probably connected with the bigger grain sizes and presence of microcracks in the walls of the material TCP-1. Scaffolds coated with P(3HB) possessed higher compressive strength (3.8 ± 0.6 MPa) and surgical maneuverability in comparison to uncoated struts. It might be due to the clogging scaffolds’ smallest micropores by P(3HB) layer. Because the open micropores and/or defects in the scaffolds were infiltrated with polymer, the open porosity decreased and the original brittle bioceramic struts were reinforced. Similar results were observed by Miao et. al. [43] who noticed an even 10-fold increase in mechanical strength of macroporous HA/TCP scaffolds coated with PLGA. Additionally, the P(3HB) microfilaments that combine ceramic cracks after the compressive test were observed (Figure 5B). If the scaffold is accidentally crushed during the surgical procedure the ceramic debris can lead to severe inflammation process. Thus, surgically handy (maneuverable) materials are of particular interest in the case of bone tissue engineering. The compressive strength values of P(3HB) coated Ag-βTCP scaffolds, obtained in this study, are sufficient to withstand the implantation procedures. The presence of pores allows for the ingrowth of new bone tissue and thus the creation of biofunctional construct with higher mechanical properties.

### 3.5. Degradation Studies and UHPLC-MS Measurements

**Figure 6:**
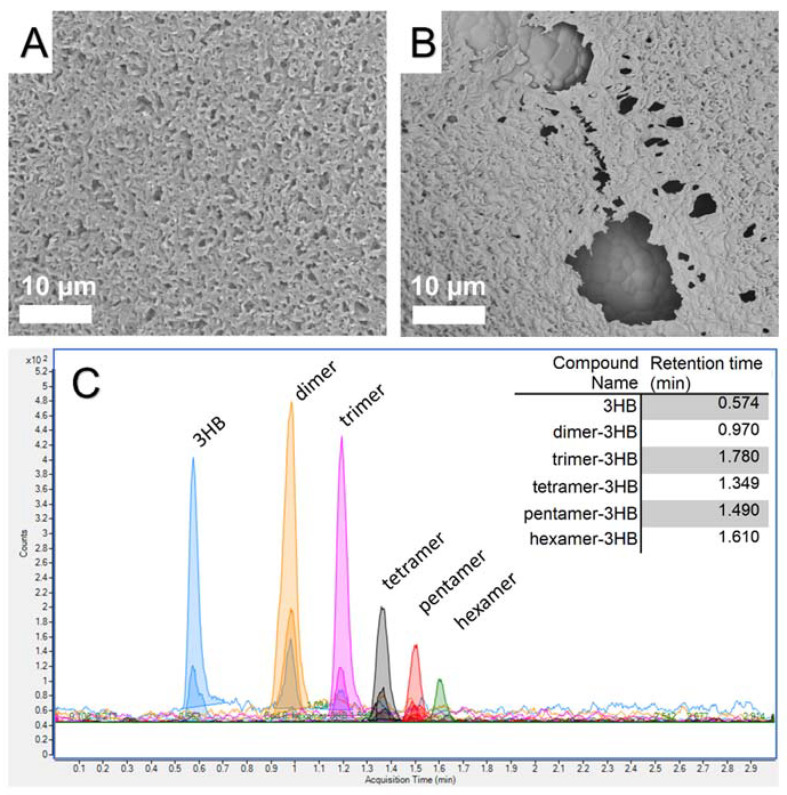
SEM microphotograph of the surface of the TCP/P(3HB) composite before (**A**) and after 120 days incubation in distilled water (**B**). The results of UHPLC-MS analysis (**C**).

TCP/P(3HB) composites were incubated in a distilled water solution for a period of 120 days. After this time, the uniform coverage by the biopolymer disintegrated through hydrolytic action of distilled water, creating voids, which revealed ceramic grains of the composite (Figure 6A vs. Figure 6B). This observation was correlated with results obtained from UHPLC-MS analysis, where the degradation components of the P(3HB) films were identified by UHPLC-qqq technique. The MS/MS analyses confirmed that the polymer degraded into not only pure (*R*)-3-hydroxybutyric acid but also to its oligomers (Figure 6C). Predominant specimens released were free acids, dimers and trimers. Further, larger oligomers were also observed: tetra-, penta- and hexamers of (*R*)-3-hydroxybutyric acid. Nevertheless, precise quantification of each oligomer was impossible, as their concentrations were below the limit of quantification of the apparatus (a level below ng·L^−1^). The slow release of P(3HB) components to the surrounding tissues over time can be beneficial. It was shown that 3-hydroxybutyrate had a stimulatory effect on cell cycle progression that is mediated by a signaling pathway-dependent upon increases in Ca^2+^ ions [44]. Thus, the combination of bioceramic with polyhydroxyalkanoate polymer support may underlie the good biocompatibility and additionally nourish surrounding tissues.

## 4. Conclusions

The macroporous β-tricalcium phosphate scaffolds modified with silver (Ag-βTCP) were prepared by a polyurethane foam replica method, followed by the coating of bacterially derived polymer-P(3HB). Materials with open porosity between 61.8 ± 3.0%–68.1 ± 4.6% and pore sizes in the range of 100–700 µm, were obtained. Concerning the consolidation of bioceramic struts, an appropriate sintering regime was achieved at 1150 °C, which is reflected in the highly dense microstructure with a small amount of microcracks. In higher temperatures, exceeding the critical grain size resulted in regions with local intergranular cracks. The compressive strength of scaffolds was in the range from 2.1 ± 0.6 to 3.8 ± 0.6 MPa, which is comparable to the compressive strength of spongy bone and can be sufficient for implantation in the low-load-bearing places. The designed scaffolds cannot be applied for load-bearing applications. Scaffolds covered with polymer possessed higher compressive strength and surgical maneuverability, sufficient to withstand the implantation procedures. Notably, in vivo the mechanical properties of scaffolds should increase with time as the tissue grows and the scaffold degrades. Moreover, releasing not only pure (*R*)-3-hydroxybutyric acid but also its oligomers during hydrolytic degradation of the composite material was confirmed, which may be beneficial for the surrounding tissues as a nourishing agent. To further improve ductility and adhesion of P(3HB) film to the scaffold it would be reasonable to try blending P(3HB) with other polymers. This would be the subject of further investigations. Further biological studies considering biocompatibility and antibacterial properties of Ag-βTCP/poly(3hydroxybutyrate) scaffolds are necessary.

## Figures and Tables

**Table 1:** The description of studied materials.

Symbol	Material	Temperature of Heat Treatment (°C)
Ag-βTCP	Ag-βTCP powder	900
TCP-1	Ag-βTCP based scaffold	1200
TCP-2		1150
TCP/P(3HB)	TCP-2 scaffold coated with P(3HB)	-

Where: TCP—tricalcium phosphate, P(3HB)—poly(3-hydroxybutyrate).

**Table 2:** The chemical composition of materials.

Chemical Element	Content (wt.%)		
	Ag-βTCP	TCP-1	TCP-2
Ca	39.351	39.276	41.062
P	18.608	17.330	17.436
O	40.262	41.570	39.535
Ag	1.097	1.259	1.127
Si	0.197	0.063	0.068
Al	0.160	0.088	0.460
Mg	0.141	0.099	0.098
Na	0.047	0.142	0.119
Fe	0.044	0.046	0.053
S	0.017	0.011	0.006
Cl	0.016	0.025	-
Sr	0.016	0.020	-

**Table 3:** The porosity of studied materials.

Material	P_total_ (vol%)	P_open_ (vol%)	P_closed_ (vol%)
TCP-1	70.8 ± 1.9	66.7 ± 2.7	4.0 ± 1.6
TCP-2	71.1 ± 2.9	68.1 ± 4.6	2.9 ± 1.1
TCP/P(3HB)	71.8 ± 1.8	61.8 ± 3.0	10.1 ± 3.0

## Data Availability

All the data is available within the manuscript.

## Supplementary Materials

The following are available online at https://www.mdpi.com/article/10.3390/ma14154227/s1, Table S1: The transitions, polarity, fragmentor and collision energies.
