# Supplementary material for: Silver Decorated βTCP-Poly(3hydroxybutyrate) Scaffolds for Bone Tissue Engineering
Source: Materials (Basel). 2021 Jul 28;14(15):4227. doi: 10.3390/ma14154227 (PMC8346965; doi:10.3390/ma14154227)
Supplement: Supplementary file 1 [file materials-14-04227-s001.zip › materials-1293158-supplementary.pdf]

materials MDPI

Supplementary Materials

Silver Decorated βTCP-poly(3hydroxybutyrate) Scaffolds for Bone Tissue Engineering

Joanna Czechowska [1,*], Szymon Skibiński [1,*], Maciej Guzik [2] and Aneta Zima [1]

[1] Department of Ceramics and Refractories, Faculty of Materials Science and Ceramics, AGH University of Science and Technology, Mickiewicza Av. 30, 30-059 Krakow, Poland; azima@agh.edu.pl
[2] Jerzy Haber Institute of Catalysis and Surface Chemistry Polish Academy of Sciences, Niezapominajek 8, 30-239 Krakow, Poland; maciej.guzik@ikifp.edu.pl
* Correspondence: jczech@agh.edu.pl (J.C.), skibinski@agh.edu.pl (S.S.)

Table S1. The transitions, polarity, fragmentor and collision energies.

Compound Name	Precursor Ion	Product Ion	Fragmentor (V)	Collision Energy (V)	Cell Accelerator Voltage (V)	Polarity
3HB	103.0	59.1	72	10	4	Negative
3HB	103.0	41.1	72	26	4	Negative
dimer-3HB	189.1	103.1	67	10	4	Negative
dimer-3HB	189.1	59.1	67	18	4	Negative
dimer-3HB	189.1	41.1	67	58	4	Negative
trimer-3HB	275.1	150.7	82	18	4	Negative
trimer-3HB	275.1	103.1	82	10	4	Negative
trimer-3HB	275.1	59.1	82	34	4	Negative
trimer-3HB	275.1	41.0	82	90	4	Negative
tetramer-3HB	361.1	228.4	97	18	4	Negative
tetramer-3HB	361.1	189.0	97	10	4	Negative
tetramer-3HB	361.1	103.1	97	14	4	Negative
tetramer-3HB	361.1	59.1	97	46	4	Negative
pentamer-3HB	447.1	189.0	102	10	4	Negative
pentamer-3HB	447.1	103.1	102	22	4	Negative
pentamer-3HB	447.1	85.2	102	26	4	Negative
pentamer-3HB	447.1	59.1	102	66	4	Negative
hexamer-3HB	533.2	314.0	102	42	4	Negative
hexamer-3HB	533.2	301.0	102	18	4	Negative
hexamer-3HB	533.2	103.1	102	30	4	Negative
heptamer-3HB	619.2	349.3	97	46	4	Negative
